# Factors affecting cervical cancer screening uptake, visual inspection with acetic acid positivity and its predictors among women attending cervical cancer screening service in Addis Ababa, Ethiopia

**DOI:** 10.1186/s12905-020-01008-3

**Published:** 2020-07-16

**Authors:** Atalay Mulu Fentie, Tamir Birhanu Tadesse, Gebremedhin Beedemariam Gebretekle

**Affiliations:** 1 Social and Administrative Pharmacy Unit, School of Pharmacy, College of Health Sciences, Addis Ababa University, P.O.Box: 1176, Addis Ababa, Ethiopia; 2 Addis Ababa Health Bureau, Addis Ababa, Ethiopia

**Keywords:** Cervical cancer, Screening uptake, VIA positivity, VIA test

## Abstract

**Background:**

Cervical cancer is the second most common cancer in Ethiopia next to breast cancer. Despite the high burden of the disease and availability of free screening services in Ethiopia, uptake is still trivial. This study aims to identify factors associated with cervical cancer screening uptake, VIA (visual inspection with acetic acid) positivity and its predictors among women attending cervical cancer screening service in Addis Ababa, Ethiopia.

**Methods:**

Concurrent mixed study approach of qualitative interview (n = 15) and cross-sectional study among 844 screened women was conducted from February to July 2018. A multistage sampling technique was employed to recruit survey participants from the selected health facilities while the key informants for an in-depth interview were selected purposively. Descriptive statistics were used to summarize the quantitative data and multivariable logistic regression was employed to explore factors associated with VIA positivity of the cervix among screened women. Qualitative data were analyzed using thematic analysis approach.

**Results:**

The VIA positivity of the cervix was 10.3%. Mean age of study participants was 35.74 ± 7.6 years and women in the age group of ≥45 years were about > 8 times more likely to have VIA test positive result compared to younger women (≤24 years). Being single (AOR = 3.2, 95%CI: 1.4–7.31), widowed (AOR = 18.6, 95%CI: 3.8–91.2), initiating sexual intercourse early (< 16 years) (AOR = 2.72; 95%CI: 1.65–4.49), and having two or more lifetime sexual partners (AOR = 4.9; 95% CI: 1.31–8.75) were also found to be predictors of being VIA positive. Lack of awareness, inaccessibility of the screening service, cultural beliefs and negative perception towards cancer were found to be the major reasons for low uptake of cervical cancer screening.

**Conclusion:**

The VIA positivity among screened women in Addis Ababa was found to be moderately low compared to reports in other parts of Ethiopia. Having multiple sexual partners, being older age and initiation of sexual intercourse at an early age were associated with VIA positivity of the cervix. Thus, concerted efforts must be taken to increase accessibility of screening services and improve awareness regarding cervical cancer screening.

## Background

Cervical cancer is a global significant public health problem; especially in low-income countries, where it is the second most commonly diagnosed cancer and third leading cause of cancer-related deaths in women. Globally, it is the fourth common cancer accounting for 6.6% of all female cancers [1, 2]. About 85% of the global burden and 87% of deaths secondary to cervical cancer occur in the less developed regions. East African region was the top in cervical cancer with age-standardized incidence of 42.7 per 100,000 population and mortality rate of 27.6 per 100,000 deaths [3]. In Ethiopia, cervical cancer is the second most common female cancer with an age-standardized incidence rate estimated in 2018 of 18.9/100,000 (about 6294 new cases and 4884 deaths estimated annually) [4, 5].

Considering its increasing burden, recently Ethiopia has put in place a strategic goal to reduce cancer incidence and mortality by 15% by 2020. This was in alignment with the 5 years National Health Sector Transformation Plan of 2016–2020 which aimed to increase awareness by 50, 80% coverage of vaccination against Human Papilloma Virus (HPV) and 80% screening coverage using visual inspection with acetic acid (VIA) testing among non-symptomatic women aged early 30–49 years. Like other resource limited settings, cervical cancer screening strategy of Ethiopia preferred VIA screening method in detecting precancerous cervical lesions. The VIA test method was chosen since it does not need more advanced testing requirements (e.g. trained cytotechnicians or pathologists and other programmatic requirements) as compared to HPV test and cytologic or Pap smear. As per the guideline, abnormalities are identified by inspection of the cervix without magnification after application of dilute acetic acid (vinegar). When vinegar is applied to abnormal cervical tissue, it temporarily turns to white (aceto-white) allowing the provider to make an immediate assessment of a positive (abnormal) or negative (normal) result [6].. The VIA test has been also shown to have an average sensitivity of 77% (ranged from 56 to 94%) and specificity of 86% (ranged from 74 to 94%) to detect precancerous lesions and cancer of the cervix. However, the major limitation of VIA test is its use in older women. Visual tests cannot be relied on in postmenopausal women, because the transformation zone of these women is often inside the cervical canal [7, 8].

To achieved Ethiopia’s 2020 ambitious goals, cervical cancer screening services are being provided free for all eligible women since 2016 [6]. Despite the availability of guideline for cervical cancer prevention and control; screening was not fully implemented in all health care centers [4] and its uptake among the community is still very low (7.3 to 23.5%) [9–15]. This could be attributed to lack of awareness about the importance and availability of cervical cancer screening services, perception about cancer, risk factors and prevention methods [12, 15–19]. On the other hand, screening uptakes as well as knowledge about cervical cancer risk factors and preventions are low among the health workers [20]. Hence, our study aimed to identify factors associated with uptake of cervical cancer screening and VIA positivity among screened women. Findings of the study could be used to inform public policy, develop and implement strategies to increase screening uptake and thereby contribute in reducing the high morbidity and mortality of the disease in the country.

## Methods

### Study area

The study was conducted in Addis Ababa, the capital city of Ethiopia and headquarters of Africa Union. It is also the largest city with population size of 7 million where male to female ratio is 99:100. Number of females in the reproductive age group (15–49 years) constitutes 35.5% of the total population. Administratively, Addis Ababa has 10 sub-cities with a total of 96 health centers and 43 hospitals (12 public and 31 private). The general health services coverage in Addis Ababa with regard to geographical accessibility is almost 100% [6].

### Study design and population

A concurrent mixed study approach of both quantitative and qualitative methods was conducted from February 1 to July 31, 2018. A cross-sectional study was carried out to determine the prevalence and factors associated with VIA positivity among women who had been screened for cervical cancer in the selected health facilities. The qualitative interview was carried out with women in the community (screened and not screened) to explore factors affecting screening uptake.

### Participants’ recruitment

**Fig. 1:**
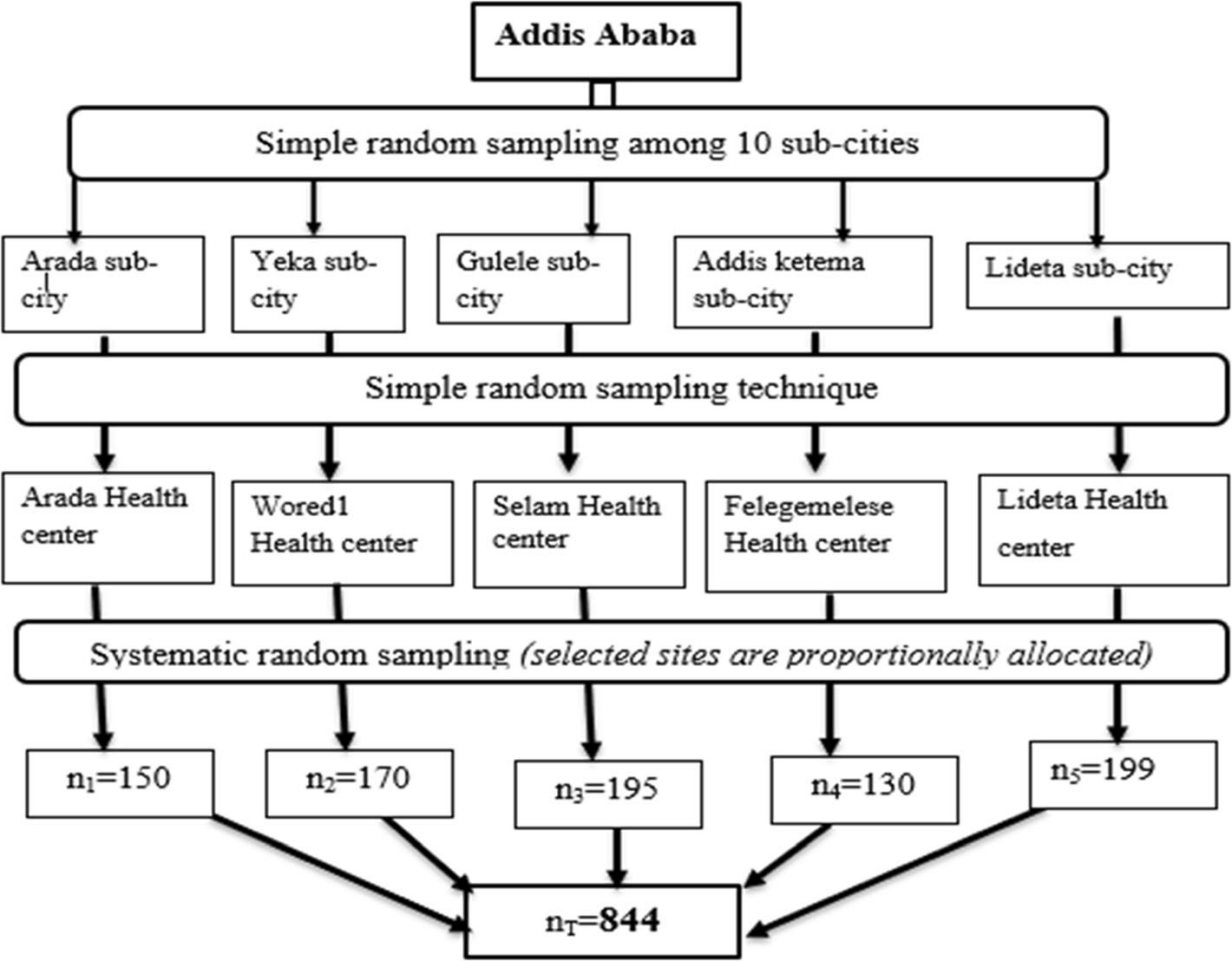
Flow chart of participant enrollment for the quantitative surveys

Among 139 healthcare facilities, cervical cancer screening service was rendered in 25 health centers and five hospitals. Of these, health centers perform only VIA test for screening. But hospitals are doing Pap smear to further investigate referred women with VIA test positive. Hence, all of our participants were recruited from health centers using a multistage random sampling technique. From the 10 sub-cities, five sub-cities and one health center from each sub-city were selected using simple random sampling technique. The total numbers of women screened in the selected health centers were 1200 and of these, we included 844 women. Sample size was calculated using a formula for single population proportion which was calculated to be 384 [21]. Then, considering a 10% inappropriate response and design effect of two, we included a total of 844. Proportional allocation of survey participants was done among the selected facilities (Fig. 1) and participants were drawn using a systematic random sampling technique.

For the qualitative interview, a purposive sampling technique was employed to select the key informants. We recruited all interviewees by consulting the health community workers who have been tasked with creating awareness and increasing uptake of the screening program. We included women who refused to be screened, not yet visited for screening and those who had been screened for cervical cancer.

### Data collection procedures and instruments

For the quantitative survey, data were collected from the screening registration logbook using a specifically designed form to capture participant’s socio-demographic characteristics (age, marital status and residence), reproductive characteristics (menstrual history, pregnancy status and parity, contraceptive use history and type of contraceptive used, family history of cervical cancer, HIV status and STI history), behavioral characteristics (age at first sexual intercourse, lifetime sexual partners, smoking habit and previous history of cervical cancer screening) and screening test result. The survey form was developed based on screening registration logbook developed by our ministry of health (Additional file 1). Data were collected by 10 trained nurses.

For the qualitative interview, we used a semi-structured open interview guide with flexible probing technique (Additional file 1). It was initially developed by the research team in English, translated to Amharic (local and national language) and back-translated into English to ensure consistency. Participants were encouraged to speak and express their ideas freely and describe their experiences with cases related to the topic. All interviews were conducted by author TB in Amharic, tape-recorded, and translated and transcribed verbatim on the same day of the interview. Interviews of participants continued until saturation was reached, meaning the investigator agreed that there was redundancy in the responses and there was no new idea emerging.

### Data analysis

Quantitative data were entered and analyzed using SPSS version 24. Descriptive statistics such as proportion, mean and standard deviation were used to summarize participants’ characteristics. Associations between the outcome variable (status of cervical lesions using VIA test) and explanatory variables were tested using multivariable logistic regression. A *p*-value of ≤0.25 was taken as a cutoff point for selecting variables for the multivariable logistic regression model. The results of all the logistic regression analysis are reported as odds ratios (OR) with 95% confidence intervals (CIs). Chi-square test was also performed to see association between health centers and VIA positivity, sociodemographic, sexual and reproductive characteristics. The VIA positivity differences between clusters (health centers) were tested using generalized linear mixed (melogit) model for binary logistic regression analysis. As per the melogit model, the between group variance value was computed using the statistical package for complex survey data (STATA 14.1). Then intra cluster correlation coefficient (ICC) for a categorical outcome was calculated using the following formula [22].

$$ \mathrm{ICC}=\mathrm{Between}\ \mathrm{group}\ \mathrm{variance}/\left(\mathrm{Between}\ \mathrm{group}\ \mathrm{variance}+{\boldsymbol{\uppi}}^2/3\right) $$

Qualitative data were analyzed manually using thematic analysis. Each interview was read thoroughly text by text and codes were labeled. Then, codes were categorized into different categories and finally, themes were formulated. The qualitative finding was shared with seven of the interviewees to confirm if interpretation was reflective of their perception and/or experiences.

## Results

### Results of quantitative survey

#### Socio-demographic and behavioral characteristics of survey participants

The mean age of the participants was 35.7 ± 7.6 years and majorities (75.8%) of them were in the age group of 26–40 years. Almost all (99.4%) of the women were living in Addis Ababa and 696 (82.5%) of them were married. With regard to their age at first sexual intercourse, majorities (69.2%) of them had first sexual intercourse at the age between 16 to 24 years. Only very few (1.3%) of them had smoking history but about two-third (66%) had multiple sexual partners. None of the participants had previous history of cervical cancer screening (Table 1).

**Table 1:** Socio-demographic and behavioral characteristics of women screened for cervical cancer, Addis Ababa, Ethiopia

Variables, n = 844	n(%)	Name of health center				
		AH	W1H	SH	FMH	LH
Age (in years), *p* = 0.008						
15–24	54 (6.4)	19 (12.7)	9 (5.3)	6 (3.1)	9 (6.9)	11 (5.5)
25–34	343 (40.7)	48 (32.0)	70 (41.2)	94 (48.2)	55 (42.3)	76 (38.2)
35–45	359 (42.5)	61 (40.7)	69 (40.6)	76 (39.0)	61 (46.9)	92 (46.2)
46–55	77 (9.1)	20 (13.3)	19 (11.2)	18 (9.2)	3 (2.3)	17 (8.6)
> 55	11 (1.3)	2 (1.3)	3 (1.7)	1 (0.5)	2 (1.6)	3 (1.5)
Residence, *p* = 0.410						
Addis Ababa (AA)	841 (99.4)	149 (99.3)	170 (100.0)	194 (99.5)	130 (100.0)	198 (99.5)
Outside AA	3 (0.6)	1 (0.7)	0 (0)	1 (0.5)	0 (0)	1 (0.5)
Marital status, *p* = 0.02						
Single	87 (10.3)	30 (20.0)	21 (12.4)	12 (6.2)	5 (3.8)	19 (9.5)
Married	696 (82.5)	112 (74.7)	141 (82.9)	169 (86.6)	111 (85.4)	163 (81.9)
Divorced	53 (6.3)	8 (5.3)	7 (4.1)	12 (6.2)	12 (9.2)	14 (7.0)
Widowed	8 (0.9)	0 (0)	1 (0.6)	2 (1.0)	2 (1.6)	3 (1.6)
Ever habit of smoking, *p* = 0.653						
Yes	11 (1.3)	3 (2.0)	2 (1.2)	3 (1.5)	0 (0)	3 (1.5)
No	833 (98.7)	147 (98.0)	168 (98.8)	192 (98.5)	130 (100.0)	196 (98.5)
Age at first sexual intercourse, *p* < 0.0001						
≤ 15	174 (20.6)	22 (14.7)	21 (12.4)	40 (20.5)	17 (13.1)	49 (24.6)
16–24	584 (69.2)	103 (68.7)	97 (57.1)	118 (60.5)	68 (52.3)	126 (63.3)
25–34	81 (9.6)	25 (16.6)	51 (30.0)	35 (17.9)	45 (34.6)	23 (11.6)
35–44	5 (0.6)	0 (0)	1 (0.5)	2 (1.1)	0 (0)	1 (0.5)
Life time sexual partners, *p* < 0.0001						
1	287 (34.0)	56 (37.3)	74 (43.5)	57 (29.2)	52 (40.0)	48 (24.1)
≥ 2	557 (66.0)	94 (62.7)	96 (56.5)	138 (70.8)	78 (60.0)	151 (75.9)
Previously screened for cervical cancer						
Yes	0 (0.0)	0 (0)	0 (0)	0 (0)	0 (0)	0 (0)
No	844 (100)	150 (100)	170 (100)	195 (100)	130 (100)	199 (100)

*p*-value is as per chi-square test done between demographic characteristics and health center type, *AH* Arada health center, *W1H* Woreda 1 health center, *SH* Selam health center, *FH* Felegemelese health center, *LH* Lideta health center.

### Sexual and reproductive history

The majorities (85.9%) of women had at least one live birth in their lifetime and most (68.1%) of them gave 1 to 3 live births. About one-third (34.4%) of the women had irregular menstrual cycles. The bulks (66.5%) of the participants never used any type of contraceptive methods. Of those who ever used contraceptives, 102(36.2%) of them used implants. Only 5(0.6%) of the women had family history of cervical cancer and 252(29.9%) of them were HIV-positive. Two hundred forty-seven (29.3%) women had at least one time sexually transmitted infection (STI) history. From the 844 screened women, 87(10.3%) of them were detected to be VIA positive indicating the presence of precancerous lesions of the cervix (Table 2).

**Table 2:** Sexual and reproductive history of women screened for cervical cancer and proportion of VIA positivity, Addis Ababa, Ethiopia

Variable, n = 844	n(%)	Name of health center				
		AH	W1H	SH	FMH	LH
Menstrual History, *p* = 0.011						
Regularly	382 (45.3)	74 (49.3)	77 (45.3)	92 (47.2)	57 (43.8)	82 (41.2)
Irregular	290 (34.4)	49 (32.7)	64 (37.6)	63 (32.3)	55 (42.3)	59 (29.6)
Post coital spotting	7 (0.8)	0 (0)	4 (2.4)	1 (0.5)	1 (0.8)	1 (0.5)
Menopause	165 (19.5)	27 (18.0)	25 (14.7)	39 (20.0)	17 (13.1)	57 (28.6)
Pregnancy status, *p* = 0.896						
Pregnant	1 (0.1)	0 (0)	0 (0)	1 (0.5)	0 (0)	0 (0)
Non-pregnant	843 (99.9)	150 (100)	170 (100)	194 (99.5)	130 (100)	199 (100)
Parity, *p* = 0.004						
No	119 (14.1)	31 (20.7)	28 (16.5)	25 (12.8)	6 (4.6)	29 (14.6)
1–3	575 (68.1)	89 (59.3)	99 (58.2)	136 (69.7)	115 (88.5)	136 (68.3)
4–5	107 (12.7)	24 (16.0)	32 (18.8)	21 (10.8)	4 (3.1)	26 (13.1)
> 5	43 (5.1)	6 (4.0)	11 (6.5)	13 (6.7)	5 (3.8)	8 (4.0)
Ever use of contraceptive methods, *p* = 0.009						
No	561 (66.5)	107 (71.3)	117 (68.8)	129 (66.2)	69 (53.1)	139 (69.8)
Yes	283 (33.5)	43 (28.7)	53 (31.2)	66 (33.8)	61 (46.9)	60 (30.2)
Type of contraceptive method, *p* = 0.317						
Injectable	86 (30.4)	12 (27.9)	14 (26.9)	22 (33.3)	20 (33.3)	18 (30.0)
OCP	42 (14.8)	10 (23.4)	8 (15.4)	11 (16.7)	5 (8.4)	6 (10.0)
IUCD	45 (15.9)	8 (18.6)	10 (19.2)	11 (16.7)	3 (5.0)	13 (21.7)
Implant	102 (36.0)	11 (25.6)	19 (36.5)	19 (28.8)	30 (50.0)	23 (38.3)
Tubaligation	1 (0.4)	0 (0)	0 (0)	1 (1.5)	0 (0)	0 (0)
Condom	7 (2.5)	2 (4.5)	1 (1.9)	2 (3.0)	2 (3.3)	0 (0)
Other corticosteroid use history, *p* = 0.556						
Yes	2 (0.2)	1 (0.7)	0 (0)	0 (0)	1 (0.8)	0 (0)
No	842 (99.8)	149 (99.3)	170 (100)	195 (100)	129 (99.2)	199 (100)
Family hx of cervical cancer, *p* = 0.687						
Yes	5 (0.6)	2 (1.3)	1 (0.6)	1 (0.5)	0 (0)	1 (0.5)
No	839 (99.4)	148 (98.7)	169 (99.4)	194 (99.5)	130 (100)	198 (99.5)
HIV status, *p* < 0.0001						
HIV positive	252 (29.9)	64 (42.7)	56 (32.9)	35 (26.9)	53 (27.2)	46 (23.1)
HIV negative	447 (53.0)	47 (31.3)	81 (47.6)	48 (36.9)	124 (63.6)	146 (73.4)
Unknown	145 (17.1)	39 (26)	33 (19.4)	47 (36.2)	18 (9.2)	7 (3.5)
STI history						
Yes	247 (29.3)	16 (10.7)	44 (25.9)	55 (28.2)	65 (50.0)	67 (33.7)
No	597 (70.7)	134 (89.3)	126 (74.1)	140 (71.8)	65 (50.0)	132 (66.3)
VIA test result, *p* < 0.0001						
Positive	87 (10.3)	8 (5.3))	8 (4.7)	30 (25.4)	11 (8.5))	30 (25.1)
Negative	757 (89.7)	142 (94.7	162 (95.3)	165 (84.6)	119 (91.5	169 (84.9)

*p*-value is as per chi-square test done between sexual and reproductive characteristics; and health center type, *AH* Arada health center, *W1H* Woreda 1 health center, *SH* Selam health center, *FH* Felegemelese health center, *LH* Lideta health center, *OCP* Oral contraceptives, *IUCD* Intrauterine contraceptive device.

### Factors associated VIA positivity among women’s screened for cervical cancer

Logistic regression analysis showed that women’s age, marital status, age at first sexual intercourse, lifetime sexual partners and HIV status were significantly associated with the rate of positive VIA test result. Screened women aged 45to 54 years (AOR = 8.1; 95%CI: 1.53–42.3) and ≥ 55 years (AOR = 8.4; 95%CI: 1.1–72.3) were more than eight times more likely to have cervical lesions as per VIA test compared to younger counterparts (≤ 24 years). Being single (AOR = 3.2, 95%CI: 1.4–7.31) and widowed (AOR = 18.6; 95%CI: 3.8–91.2) were also found to be predictors of having cervical lesions as per VIA test. Women who started sexual intercourse before the age of 16 years were about three times (AOR = 2.72; 95% CI: 1.65–4.49) more likely to have cervical lesions as per VIA test than their counterparts. Moreover, women who had two or more lifetime sexual partners were significantly associated with cervical lesions (AOR = 4.9; 95% CI: 1.31–8.75). In addition, HIV positive women (AOR = 2.59, 95%CI: 1.56–4.25) were more likely to have cervical lesions compared to HIV negative women (Table 3). Given that small numbers of VIA positive results (87 VIA positive cases out of 844 screened women) and differences with respect to sociodemographic, sexual and reproductive characteristics (Tables 1 and 2) among health centers (clusters), we would expect much variation in the actual number of VIA positivity reported in each cluster and consequently, it is important to estimate ICC. This is shown with our estimate of ICCs (0.069) for observed VIA positivity among women screened for cervical cancer in Table 3.

**Table 3:** Univariate and multivariable logistic regression analysis results for predictors of VIA positivity among women screened for cervical lesion, Addis Ababa, Ethiopia

Variable	Screening result, n (%)		COR (95%CI)	AOR (95%CI)
	Positive	Negative		
Age (in years)				
≤ 24	2 (2.3)	52 (6.9)	1.00	1.00
25–34	27 (31.0)	316 (36.9)	2.2 (0.5–9.6)	2.4 (0.51–11.5)
35–44	40 (46.0)	319 (42.1)	3.26 (0.77–13.9)	3.8 (0.81–18.2)
45–54	15 (17.2)	62 (8.1)	6.3 (1.4–28.8)	8.1 (1.53–42.3)
≥ 55	3 (3.5)	8 (3.0)	9.8 (1.4–67.7)	8.4 (1.1–72.3)
Marital status				
Married	62 (71.3)	634 (83.8)	1.00	1.00
Single	12 (13.8)	75 (9.9)	1.6 (0.8–3.2)	3.2 (1.4–7.31)
Divorced	8 (9.2)	45 (5.9)	1.8 (0.8–4.1)	1.8 (0.76–4.27)
Widowed	5 (5.7)	3 (0.4)	17.1 (4.1–72.9)	18.6 (3.8–91.2)
Parity				
Null Para	11 (12.6)	108 (14.3)	1.00	1.00
Multi Para	64 (73.6)	568 (75.0)	1.1 (0.56–2.2)	1.7 (0.72–4.0)
Grand multi Para	12 (13.8)	81 (10.7)	1.46 (0.61–3.5)	1.9 (0.63–5.52)
Age at first sexual intercourse				
≥ 16 years	52 (59.8)	617 (81.5)	1.00	1.00
< 16 years	35 (40.2)	140 (18.5)	3.1 (1.86–4.73)	2.72 (1.65–4.49)
Ever use of contraceptive				
No	59 (67.8)	502 (66.3)	1.00	1.00
Yes	28 (32.2)	255 (33.7)	0.93 (0.58–1.51)	0.64 (0.37–1.11)
Smoking habit history				
No	86 (98.8)	747 (98.7)	1.00	1.00
Yes	1 (1.2)	10 (11.3)	0.88 (0.11–6.87)	1.25 (0.13–11.8)
Lifetime number of sexual partners				
1	15 (17.2)	272 (35.9)	1.00	1.00
≥ 2	72 (82.8)	485 (64.1)	5.6 (1.9–9.82)	4.9 (1.31–8.75)
HIV status				
HIV negative	34 (39.1)	413 (54.6)	1.00	1.00
HIV positive	44 (50.6)	208 (27.5)	2.57 (1.59–4.14)	2.59 (1.56–4.25)
Unknown	9 (10.3)	136 (17.9)	0.80 (0.38–1.72)	0.88 (0.41–1.93)
STI history				
No	65 (74.7)	532 (70.3)	1.00	1.00
Yes	22 (25.3)	225 (29.7)	0.8 (0.48–1.33)	1.46 (0.84–2.53)
Between group variance	0.245 (95%CI: 0.0446–1.247, *p* < 0.0001)			
ICC	0.069			

*ICC* Intra-cluster correlation coefficient. The between group variance and ICC was computed to see VIA test positivity (dependent variable) variability among type of health center cluster s (independent variable) as per multilevel mixed effects models for logistic regression. ICC = 0.245/(0.245 + **π**^2^/3) = 0.245/3.535 = 0.069

### Findings of qualitative interview

In-depth interviews were conducted with 15 women their mean age was 35.8 ± 5.4 years (ranged from 23 to 55 years). Regarding parity, four of them didn’t give live birth whereas six, three and two of them had 1 to 3, 4 to 5 and > 5 live births in their lifetime, respectively. Twelve of them were married and the rest were single. We asked all women to describe the perceived factors that they believe are affecting the screening uptake of cervical cancer. Accordingly, main themes emerged as the key barriers for women to undergo cervical cancer screening were lack of awareness, inaccessibility of the screening service, cultural and religious beliefs.

### Lack of awareness

Most of the respondents were familiar with the word cervical cancer and their source of information was from neighborhoods and mass Medias such as radio and television. When they were asked about cervical cancer, however, they often referred their knowledge to cancer in general. A woman with screening history stated that:

*“I heard the word cervical cancer in television advertising and in some radio programs but I don’t have full information because the programs are too short and they didn’t transmit the full information. It is a simple advertisement and what I heard is simply a promotion to be screened for cervical cancer.”*

On the other hand, majority of the respondents lacked adequate information about the cause of cervical cancer and only two participants responded correctly that having multiple sexual partners as the cause of cervical cancer and they heard it from their neighborhoods. Many respondents perceived cancer and by extension cervical cancer as a deadly disease with no cure. They related the experiences of their friends and/or family members who died of cervical or other cancers. All stated cervical cancer; and cancer in general as a horrible disease and one that often incurred huge stress, emotions and physical suffering on both the patient and their family members.

Even though most respondents heard about cervical cancer, most of them did not know about the sign and symptoms of the disease. Absence of the symptoms was mentioned as a barrier for cervical cancer screening. The community gave priorities to diseases with significant signs and symptoms. One of the participant screened for cervical cancer stressed that;

*“I think the reason why most women are not screened for cervical cancer is mainly attributed to the nature of the disease. I mean, at that stage they are apparently healthy and had no symptom. We are more concerned if there are noticeable and/or visible signs and symptoms, which is not true for screening programs like this.”*

Most participants mentioned that there is lack of adequate information about the existence of cervical screening, eligibility for screening, screening sites and time schedule for screening. Most women heard of cervical cancer screening but they did not know where it is provided. A study participant who did not visit a health center for screening stated that:

*“I think all women in my community don’t have information about cervical cancer screening but had they known the detailed information about it, I am sure they would have been screened. Sometimes I heard radio/TV advertainments but they don’t convey detailed information about the disease, benefits and risks of screening, where and when to screen, etc.”*

Another in-depth participant added:

*“Ethiopia has a good record of community health workers but their role in cervical cancer screening is very limited. They are teaching us about hygiene, diabetes mellitus, family planning and hypertension. It would be more cost-effective if cervical cancer screening is considered as one of the packages in community training or teaching.”*

A prevailing notion among the participant of the study was cervical cancer screening is only used for the detection of cancer that is already present. Some women refused to be screened as they thought that having cervical cancer screening is not beneficial while others believed that it is a service to be given for sick women only. A study participant who refused for screening stressed that:

*“I don’t think the screening for cervical cancer is important for healthy women of my age. I think it is better to screen those women who are sick or in menopausal age […] because in most cases cervical cancer is very common in those women in my community.”*

### Cultural and religious beliefs

Some of the participants mentioned that the health professionals’ approach during the screening procedure was a barrier for screening. Participants mentioned that their culture is very sensitive towards examining female genital organ by male health professionals. This was strengthened by a screened in-depth interview participant:

*“Before one year, I was examined for cervical cancer by a male health care provider and I was so ashamed when he asked me to undress. It was very difficult to accept and I think this might be because of our culture which is so sensitive to such things.”*

Bad perception about cancer as a whole also prevented them to be screened. They tried to associate it with religious beliefs and believed that it is their GOD’s seek to prevent such types of bad things. Accordingly, some of them preferred to pray for God instead of screened and treated. They also heard and believed that the cancer treatment outcome is poor even if treated; so, it’s better not to be screen rather than screened and told having cancer. One participant said:

*“I preferred to die immediately rather than do the screening and being told having cervical cancer”.*

### Inaccessibility of screening services

Some of the participants mentioned that cervical cancer screening service was not available in their vicinity and this might be contributed to the lower screening uptake. Furthermore, quite a large number of women had no information that the service is given free of charge. One of the interviewee stated that:

*“I am not aware that the service is given freely. Most of us think that we have to pay for screening but we can’t afford it. We heard that the screening is dump expensive in private hospitals in Addis Ababa.”*

## Discussion

With the overall burden of cervical cancer projected to continue rising over the next decades, Ethiopia has introduced several initiatives to reduce its impact. Among others, cervical cancer screening program using VIA test has been implemented recently. To this effect, we assessed the VIA positivity rate among screened women and reasons for the low screening uptake. The VIA positivity rate was 10.3% which is slightly lower than the previous study done in South West Ethiopia (12.3%) [23] and Sudan (12.7%) [24], though the sample size in these studies was almost half and 1/7th of the present study, respectively. Although different study populations (HIV positive patients only), studies in Southern Ethiopia reported highest proportions of VIA positivity (22.1%) [25], which is almost similar to Tanzania (17%) [26]. The findings in the present study reveals that cervical cancer is still a significant public health problem for both HIV positive and negative women. This warns screening service provision should get special attention, knowing that early detection and treatment is more cost-effective than treating advanced disease [7].

Similar to previous studies [1, 27–29]; advanced age, having multiple sexual partners, being single or widowed, initiating first sexual intercourse at an early age and being HIV positive were found to be predictors of VIA positivity. Women aged ≥45 years had higher odds of having VIA positive test result compared to those aged ≤24 years. This is similar to studies conducted in Addis Ababa [28, 29] and Jimma [16]. All these associations have been already observed and that they are consistent with what we know of the etiology and natural history of the disease [8, 30].

Having multiple sexual partners and early age at first sexual intercourse were significantly associated with having positive VIA test results compared to their counterparts, and both factors are known to increase the chance of acquiring HPV infection [29]. Especially starting sexual intercourse at early age may increase the risk, because at young age, the cervix has an immature membrane thus making it more susceptible to ontological agents particularly high-risk type of HPV [31] and this has been substantiated by studies done elsewhere [23, 32–34]. The present study also found that being single or widowed were significantly associated with positive VIA test result compared to married women. This might be because those women may not be abstained or had a greater chance of having multiple sexual partners resulting in an increased risk of acquiring HPV infection. This calls for policymakers to devise mechanisms to improve their awareness about cervical cancer and available screening services.

Screening and early treatment of cervical cancer if detected remains the most effective way to reduce the mortality associated with the disease. But different studies including in Ethiopia showed cervical cancer screening uptake is still low, ranged from 7.3 to 23.5% [9–14, 35–37]. Concurrent to previous studies [12, 20, 35–38], our qualitative study revealed that lack of awareness or knowledge about cervical cancer and its causes, symptoms, fear of diagnosis and treatment among women decreased their participation rates in screening. On another note, women’s unwillingness for screening was attributed to cultural and religious norms. This was expressed in several forms such as better to go to holy water and pray, the lack of desire to disrobe for the pelvic examination and concerns at being examined by male healthcare providers. Some participants in this study also did not know the presence of screening services and who will be screened. Hence, as part of the primary prevention strategies for cervical cancer, screening related educational programs need to emphasize on the value of the methods, when, how and for whom.

## Conclusions

The VIA positivity among screened women was found to be 10.3%. Having multiple sexual partners, being HIV positive, marital status and early age at initiation of sexual intercourse were identified as predictors of precancerous lesions of the cervix. Women perceived that the lack of awareness regarding cervical cancer and cervical cancer screening, inaccessibility of the screening service, cultural and religious beliefs including fear of the screening procedure; were the major barriers to cervical cancer screening uptake. This calls the need to improve the knowledge and awareness of the community about the disease and screening services through the existing health extension program.

## Supplementary information

**Additional file 1.** English version of survey form and in-depth interview guide

## Data Availability

All the data and materials used in this paper are available from the corresponding author upon reasonable request.

## Abbreviations

HIV: Human immunodeficiency virus
HPV: Human papillomavirus
IUCD: Intrauterine contraceptive device
OCP: Oral contraceptives
STI: Sexually transmitted infection
VIA: Visual inspection with acetic acid
