# Supplementary material for: Factors affecting cervical cancer screening uptake, visual inspection with acetic acid positivity and its predictors among women attending cervical cancer screening service in Addis Ababa, Ethiopia
Source: BMC Womens Health. 2020 Jul 16;20:147. doi: 10.1186/s12905-020-01008-3 (PMC7366887; doi:10.1186/s12905-020-01008-3)
Supplement: Supplementary file 1 — Additional file 1. English version of survey form and in-depth interview guide [file 12905_2020_1008_MOESM1_ESM.docx]

**Regional Health Bureau of Addis Ababa, Ethiopia**

**Data abstraction form/Quantitative survey**

**Purpose of the Study:** To asses factors affecting cervical cancer screening uptake, visual inspection with acetic acid positivity and its predictors among women attending cervical cancer screening service in Addis Ababa, Ethiopia

.**Part 1 Socio demographic status**

- Age: _________years
- Residence

□ Addis Ababa; Specify sub-city: ______________________________

□ Outside Addis Ababa; Specify city/town: _______________________

- Marital status: □ Single □ Divorce □ Married □ Widowed.
- How many children does she have? __________ children
- When was the screening made? ______dd.________mm._______yy.

**Part 2: Reproductive history at the time of screening**

- Was she pregnant? □Yes □ No
- If yes, how many weeks was the pregnancy? _______________weeks
- Did she ever use any contraceptive methods? □Yes □ No
- If yes, specify the type of contraceptive: ____________________________________
- How is the frequency of her Ministerial Cycle?

□ Regular (between 23-35 days interval) □ Irregular □ Post coital spotting

- How many sexual partners does she had? _________________

**Part 3: Risk factor for cervical cancer assessment**

- Does she have a smoking habit? □Yes □ No
- What was her age at first sexual intercourse? __________ years
- Does she have any family/relative with history cervical cancer? □Yes □ No
- If yes, what was her relationship with the person? ____________________________
- What is her HIV/AIDS status? □ HIV positive □ HIV negative □Unknown
- Does she have previous history of chronic corticosteroid drug use? □Yes □ No
- Does she have previous history of abnormal Pap smear result? □Yes □ No
- What was the VIA test result? □ HIV positive □ HIV negative □Unknown

**Part IV: Result of cervical cancer screening**

- What is the finding of the cervical cancer screening?

□ Positive □ Negative

**Qualitative interview**

**English version in depth interview consent form**

**Title**: Factors affecting cervical cancer screening uptake, visual inspection with acetic acid positivity and its predictors among women attending cervical cancer screening service in Addis Ababa, Ethiopia

**Introduction**

Hallo. My name is ________________. I am inviting you to participate in this research study titled “Factors affecting cervical cancer screening uptake, visual inspection with acetic acid positivity and its predictors among women attending cervical cancer screening service in Addis Ababa, Ethiopia”. We hope that from this study we will get information necessary to provide guidance to key stakeholders on improving access to cervical cancer screening services.

The purpose of this consent form is, to give you the information you will need to help you decide whether to be in the study or not. Please read this form carefully or listen as it is read to you and ask any questions you may have before agreeing to be involved in the study.

Your cooperation and willingness for the participation is very helpful in identifying the problems related to the issue. Your name will not be written in the form and I assure you that all information that you give will be kept strictly confidential. Your participation is voluntary and you are not obliged to answer any question you don’t wish to answer.

Do you have volunteered to participate in the study? Yes No

# **English version of in**-depth interview questionnaire

.**Part 1 Socio demographic status**

- Age: _________years
- Residence: □ Addis Ababa; specify the sub-city: __________________

□ Outside Addis Ababa; Specify city/town: ____________

- Marital status: □ Single □ Divorce □ Married □ Widowed.
- How many children does she have? __________ children

When was the screening made? ______dd.________mm._______yy.

**Part 2: In-depth interview questionnaire**

1. Have you heard of about cervical cancer? If yes, from where do you hear?
2. What do you think are the causes of cervical cancer? Can you tell us the signs and symptoms?
3. Are you aware of a free cervical cancer screening program is available in Addis Ababa?
4. Did you ever screened for cervical cancer? (if the answer is NO go to question number 7)
5. How would you describe your involvement in the screening? Can you tell us negative and positive things you encountered during the screening? (Probe on key source information on positive thing they like and negative thing they dislike)
6. How do you describe the health professionals’ approach during the screening time?
7. If not ever screened for cervical cancer before can you tell us the reason? (Probe on key questions why they are not screened)
8. What do you suggest that will initiate the women involvement in the screening process?
9. How do you describe the health extension roll in the community regarding cervical cancer screening program?
10. What do you think the major barriers facing women’s in the community not to involve in the cervical cancer screening program? How can they be overcome?
11. What are the major strengths of cervical cancer screening? How can they be built on?
12. Can you describe ways that you feel could be successful in reaching the community? (Probe on key sources of information for cervical cancer screening by the community).
13. Are there any additional comments you would like to share on cervical cancer screening?
